# Correlation of bioactive components of platelet rich plasma derived from human female adult peripheral blood and umbilical cord blood with age

**DOI:** 10.1038/s41598-023-45747-3

**Published:** 2023-10-27

**Authors:** Ju Tian, Xiong Jie Li, Yongshi Ma, Zhiming Mai, Yao Yang, Min Luo, Wenping Xu, Kui Chen, Xuri Chen, Jianbing Tang, Biao Cheng, Xiao Cui

**Affiliations:** 1 Department of Plastic Surgery, People’s Hospital of Zhongshan City, Zhongshan, 528421 Guangdong China; 2 College of Life Science and Technology, Jinan University, Guangzhou, 510630 Guangdong China; 3 Obstetrics and Gynaecology Department, General Hospital of Southern Theatre Command, PLA, Guangzhou, 510010 China; 4 Guangzhou University of Chinese Medicine, Guangzhou, 510405 Guangdong China; 5 Department of General Practice, General Hospital of Southern Theatre Command, PLA, Guangzhou, 510010 China; 6 Department of General Practice, The Third Affiliated Hospital of Guangzhou Medical University, Guangzhou, 510150 China; 7 Department of Burn and Plastic Surgery, General Hospital of Southern Theatre Command, PLA, Guangzhou, 510010 China; 8 The Key Laboratory of Trauma Treatment & Tissue Repair of Tropical Area of Chinese PLA, Guangzhou, 510010 China; 9 Department of Physiotherapy, Guangdong Provincial Hospital of Chinese Medicine, Guangzhou, 510405 Guangdong China

**Keywords:** Health care, Medical research

## Abstract

Platelet-rich plasma (PRP) has gained significant attention in the field of regenerative medicine due to its potential therapeutic applications. However, few studies have reported the components, especially anti-ageing-related components, of PRP derived from umbilical cord blood (UCB). It is essential to understand the influence of age on the composition and efficacy of PRP to optimize its clinical use. The present study compared the concentrations of bioactive components in PRP from healthy female adults and UCB-derived PRP. PRP was obtained from blood samples from females in four age groups (12 per group): neonates (UCB donors) and adults aged 18–25, 26–45, and 46–65 years, respectively. The concentrations of epidermal growth factor, basic fibroblast growth factor-2 (FGF-2), insulin-like growth factor-1, platelet-derived growth factor-AA (PDGF-AA), PDGF-AB/BB, vascular endothelial growth factor A, RANTES, TIMP-1, TIMP-2, GDF11, and clusterin and activity of superoxide dismutase, catalase, and glutathione peroxidase (GPx) in the PRP samples were determined and compared among groups. Pairwise comparisons between the groups showed statistically significant differences in the concentrations of some bioactive components of PRP, such as FGF-2, PDGF-AB/BB, and clusterin, and GPx activity. UCB-derived PRP contains various active ingredients such as VEGF-A, CAT activity, and TIMP-2. Contrary to expectations, UCB-derived PRP did not show higher concentrations of the anti-ageing protein GDF11. Because UCB is a rich source of bioactive components with low immunogenicity, its use in PRP preparation is an important research direction for future studies.

## Introduction

During the last five decades, the life expectancy of humans has increased worldwide, leading to exhaustive research efforts to identify strategies and interventions to slow ageing. Platelet-rich plasma (PRP) therapy has emerged as a revolutionary treatment strategy for skin rejuvenation; it induces cell growth in the skin, thus retarding and reversing the ageing process^1^. PRP, obtained by centrifugation of whole blood^2^, contains high concentration of platelets, which release a wide range of bioactive molecules such as platelet-derived growth factor (PDGF), transforming growth factor (TGF)-β, and vascular endothelial growth factor (VEGF)^3^. In tissue injury, PRP treatment stimulates tissue regeneration, as the growth factors (GFs) and glycoproteins (adhesion factors) in platelet granules and plasma together promote cell division, growth, and differentiation^4^. Because PRP is a product of autologous origin, PRP therapy has several advantages over conventional treatment strategies (surgery or laser treatment, etc.), including no immune rejection, no cross-infection with pathogenic microorganisms, and less side effects. Moreover, PRP preparation requires less equipment and has a simpler protocol. Of the bioactive components of PRP, GDF11 protein in rodent plasma has been suggested to reverse the ageing process in the muscle and brain and have anti-ageing effects^5–7^. Although some studies have questioned the anti-aging effect of GDF11^8^, considering the high concentrations of GDF11 in platelets, we hypothesise that the anti-ageing effects of PRP depends not only on GFs but also on anti-ageing proteins.

Although PRP has been used in clinical settings for decades, its clinical effects remain debated. Several factors such as age and gender affect the release of active ingredients from PRP, and it is difficult to prepare standardized PRP with consistent quality. The regenerative ability of most tissues gradually declines with age. Lohmann et al. reported that young fibroblasts were more sensitive to PRP therapy, suggesting that the regenerative ability of platelet concentrate decreases with age^9^. It was also found that PRP therapy for facial rejuvenation and treatment of Achilles tendinitis had poor efficacy in elderly patients (60-years old)^10,11^. There are two possible explanations for this finding: the ability of tissue regeneration and repair declines in the elderly, and the quantity of bioactive substances released by PRP in the elderly differs from that in younger individuals.

Blood therapy has long been favoured in the research field of biological regeneration and anti-ageing. A study in the United States showed that the blood-derived factor TIMP-2 counters ageing-related changes better in younger mice than in older mice^12^. Human umbilical cord blood (UCB) has been reported to have a similar activating effect on ageing tissues. However, only a few studies have reported the components of UCB-derived PRP^13,14^ and the correlation between GFs, age, and sex in adult peripheral blood-derived PRP^15–19^. Hormonal differences between males and females lead to various gender-associated differences in PRP composition; in particular, female menstruation and menopause significantly affect hormone levels. Studies indicate that gender has a greater impact than age on PRP composition^20^. Our previous research investigated the impact of age on the composition and efficacy of adult PRP, but there was no detailed study on UCB derived PRP^20–22^.

UCB is often discarded after birth; however, because it is a rich source of various active substances with low immunogenicity, UCB is an ideal raw material for the preparation of PRP. Previous studies indicate that UCB PRP leads to significantly higher proliferation of mesenchymal stem cells than adult PRP^14^. Because age is an important factor affecting the active ingredients in PRP, we aimed to examine whether PRP derived from UCB has a higher concentration of active ingredients than PRP derived from peripheral blood. To exclude the effect of gender, we derived PRP only from female UCB and peripheral blood obtained from adult females and analysed the differences in active ingredients of PRP among the various age groups. The study aimed to determine the concentrations of bioactive components, especially anti-ageing-related substances, of PRP from healthy female adults of different ages and examine whether they differ significantly from those in UCB-derived PRP.

## Methods

### Ethical approval

The present study was conducted in accordance with the 1975 Declaration of Helsinki and with approval from the Ethics Committee of General Hospital of Southern Theatre Command (Guangdong, China). Prior to the investigations, screening, and blood drawing, all participants signed an informed consent form, which explained the purpose of the study and allowed them to decline participation without affecting their ability to donate blood.

### Participant selection

In total, 48 donor individuals were enrolled in the study and divided into four groups according to their age (12 individuals per group). Group A included healthy female neonates (UCB donors) born in our hospital (0.74 ± 0.03 years); the gestational age ranged between 35 to 40 weeks. The exclusion criteria for group A was pregnant women with immune thrombocytopenia or a history of diabetes mellitus.

The other three groups consisted of healthy adult females: Group B: age 18–25 years (22.00 ± 2.09 years), Group C: 26–45 years (36.92 ± 6.65 years), and Group D: 46–65 years (58.25 ± 6.00 years). The inclusion criteria for groups B, C, and D were as follows: healthy women aged 18–65 years; blood pressure, pulse, and body temperature within the normal range; and normal blood routine and liver and kidney function tests. The exclusion criteria were as follows: smokers; individuals with a history of blood transfusion; pregnant or menstruating individuals; individuals with tumours, infectious diseases, blood system diseases, and other diseases that may affect platelet number or function.

### Sample collection and platelet-rich plasma preparation

20 mL of UCB or peripheral blood was drawn in a sterile syringe containing ethylenediaminetetraacetic acid (an anticoagulant) and centrifuged at 377×*g* for 5 min at room temperature (25°) to separate erythrocytes from the plasma with the buffy coat at the interface. Next, the upper plasma layer and buffy coat were collected and centrifuged at 1500×*g* for 15 min at room temperature. Then, the concentration of platelet in PRP was adjusted to 1000 × 10^9^/L according to our previous method^22^. PRP was activated by adding 0.1 mL of the activator (thrombin and calcium gluconate; 1:1) for every 1 mL of PRP solution. Samples were immediately snap frozen in liquid nitrogen and stored at − 80 °C.

### Haematological analysis

Platelets and white blood cells (WBC) in whole blood samples were counted using an automated Sysmex hematology analyzer (model XS800 I, Japan).

### Materials

Automated hematology analyzer (Sysmex hematology analyzer, model XS800 I, Japan). Multiplex, bead based, immunoassay system (Millipore Inc, Billerica, MA, USA). ELISA kits (IGF-1, GDF11, and clusterin) Ray Biotech (Norcross, GA). Superoxide Dismutase Assay Kit (Cayman Chemical Company, Ann Arbor, MI, USA), Catalase Assay Kit (Cayman Chemical Company, Ann Arbor, MI, USA) and Glutathione Peroxidase Activity Colorimetric Assay Kit (BioVision, Milpitas, CA, USA).

### Quantification of bioactive components

Aliquots were thawed no more than once to minimize degradation of bioactive substances. The concentrations of epidermal growth factor (EGF), basic fibroblast growth factor-2 (FGF-2), platelet-derived growth factor-AA (PDGF-AA), PDGF-AB/BB, vascular endothelial growth factor A (VEGF-A), RANTES, TIMP-1, and TIMP-2 in PRP were determined using a multiplex, bead-based, immunoassay system (Millipore Inc, Billerica, MA, USA). The concentrations were calculated using a four- or five-parameter standard curve, and samples were assayed in duplicate and averaged to calculate the concentrations. The concentrations of insulin-like growth factor-1 (IGF-1), GDF11, and clusterin in the PRP samples were determined using ELISA kits (Ray Biotech, Norcross, GA, USA). The activity of superoxide dismutase (SOD), catalase (CAT), and glutathione peroxidase (GPx) in PRP was measured using the Superoxide Dismutase Assay Kit (Cayman Chemical, Ann Arbor, MI, USA), Catalase Assay Kit (Cayman Chemical) and Glutathione Peroxidase Activity Colorimetric Assay Kit (BioVision Inc., Milpitas, CA, USA), respectively. Each sample was tested in duplicate, and each experiment was repeated at least three times.

### Statistical analysis

Multiple comparisons were performed using post-hoc Fisher least significant difference (LSD) analysis. Pearson’s correlation coefficient was used to analyse the correlation of participant age with 14 analytes. GraphPad Prism 7.0 (La Jolla, CA, USA) was used to generate all charts. Statistical procedures were performed using Microsoft Excel software and IBM SPSS Statistics 24. *P* < 0.05 was set as the statistical significance level in all tests.

## Results

### Analysis of levels of different bioactive substances among groups

The results showed that except for IGF-1 and RANTES, the concentrations of other components were statistically different among different groups (Table 1). Group A showed significantly higher concentrations of VEGF-A (366.07 ± 395.04 pg/mL) and TIMP-2 (45,927.17 ± 6195.48 pg/mL) and significantly higher CAT activity (167.71 ± 61.00 nmol/min/mL; Table 1) than other groups. Group B showed significantly higher concentrations of EGF (560.72 ± 180.80 pg/mL), PDGF-AA (12,291.33 ± 3313.06 pg/mL), and TIMP-1 (139,087.08 ± 28,465.14 pg/mL) and significantly higher SOD activity (6.20 ± 3.16 U/mL; Table 1) than other groups. Group C showed significantly higher concentrations of FGF-2 (251.56 ± 195.07 pg/mL), PDGF-AA/AB (16,185.5 ± 8000.58 pg/mL), GDF11 (202.47 ± 122.57 pg/mL), and clusterin (98.38 ± 60.24 pg/mL) and significantly higher GPx activity (118.75 ± 17.02 nmol/min/mL; Table 1). VEGF-A and GDF-11 levels in Group D were the lowest among all groups.

**Table 1:** Correlation between the age of umbilical cord blood or peripheral blood donors with the concentration or activity of 14 analytes in platelet-rich plasma.

Variable	Group A (*n* = 12)	Group B (*n* = 12)	Group C (*n* = 12)	Group D (*n* = 12)	*P* values for pairwise comparison^#^					
					A vs. B	A vs. C	A vs. D	B vs. C	B vs. D	C vs. D
Age (years)	0.74 ± 0.03	22.00 ± 2.09	36.92 ± 6.65	58.25 ± 6.00	0.00**	0.00**	0.00**	0.00**	0.00**	0.00**
PLT (× 109/L)	337.67 ± 60.780	307.5 ± 61.78	214.92 ± 32.38	261.83 ± 56.09	0.179	0.000**	0.001**	0.000**	0.045*	0.039*
WBC (× 109/L)	6.48 ± 1.99	6.59 ± 1.62	6.82 ± 3.17	5.67 ± 1.59	0.013*	0.021*	0.001**	0.837	0.411	0.306
IGF-1 (pg/mL)	19,268.75 ± 51,917.99	2241.83 ± 2517.11	324.26 ± 450.62	629.78 ± 1150.60	0.116	0.081	0.086	0.857	0.880	0.977
EGF (pg/mL)	300.11 ± 237.66	560.72 ± 180.80	212.62 ± 105.22	218.54 ± 142.56	0.001**	0.224	0.256	0.000**	0.000**	0.934
FGF-2 (pg/mL)	62.69 ± 47.37	168.42 ± 102.17	251.56 ± 195.07	174.53 ± 76.31	0.035*	0.000**	0.026*	0.094	0.900	0.120
VEGF-A (pg/mL)	366.07 ± 395.04	280.68 ± 222.42	215.41 ± 80.09	109.83 ± 47.49	0.371	0.118	0.009**	0.493	0.077	0.270
PDGF-AA (pg/mL)	5875.28 ± 3005.83	12,291.33 ± 3313.06	8727.17 ± 3596.95	6260.4167 ± 1499.04	0.000**	0.023*	0.752	0.005**	0.000**	0.048*
PDGF-AB/BB (pg/mL)	1257.79 ± 595.33	8377.75 ± 2467.81	16,185.5 ± 8000.58	8494.67 ± 3588.78	0.000**	0.000**	0.000**	0.000**	0.950	0.000**
RANTES (pg/mL)	125,366.58 ± 85,548.04	184,753.75 ± 51,755.59	154,906.83 ± 49,305.29	130,228.1667 ± 72,719.14	0.034*	0.283	0.859	0.278	0.051	0.369
TIMP-1 (pg/mL)	89,911.33 ± 32,069.20	139,087.08 ± 28,465.14	90,831.25 ± 14,181.49	109,439.92 ± 20,915.35	0.000**	0.928	0.061	0.000**	0.006**	0.074
TIMP-2 (pg/mL)	45,927.17 ± 6195.48	45,186.08 ± 5154.36	38,664 ± 2718.60	41,302 ± 4097.44	0.702	0.000**	0.021*	0.002**	0.050	0.178
GDF11 (pg/mL)	128.47 ± 70.91	143.33 ± 101.94	202.47 ± 122.57	85.18 ± 38.45	0.686	0.049*	0.242	0.112	0.118	0.002**
Clusterin (pg/mL)	13.35 ± 5.44	21.29 ± 4.67	98.38 ± 60.24	73.39 ± 36.40	0.585	0.000**	0.000**	0.000**	0.001**	0.090
SOD activity (U/mL)	5.65 ± 2.71	6.20 ± 3.16	0.19 ± 0.04	0.52 ± 1.11	0.534	0.000**	0.000**	0.000**	0.000**	0.709
GPx activity (nmol/min/mL)	19.98 ± 4.07	34.0 ± 15.68	118.75 ± 17.02	104.32 ± 29.89	0.076	0.000**	0.000**	0.000**	0.000**	0.070
CAT activity (nmol/min/mL)	167.71 ± 61.00	136.21 ± 36.74	51.38 ± 7.06	60.50 ± 20.39	0.044*	0.000**	0.000**	0.000**	0.000**	0.551

Values in this table are shown as mean ± standard deviation.

*PLT* platelets, *WBC* white blood cells.

^#^Fisher’s LSD analysis was used for multiple comparisons.

*Represents *P* < 0.05.

**Represents *P* < 0.01.

### Relationship between bioactive components concentration in PRP and female age

**Figure 1:**
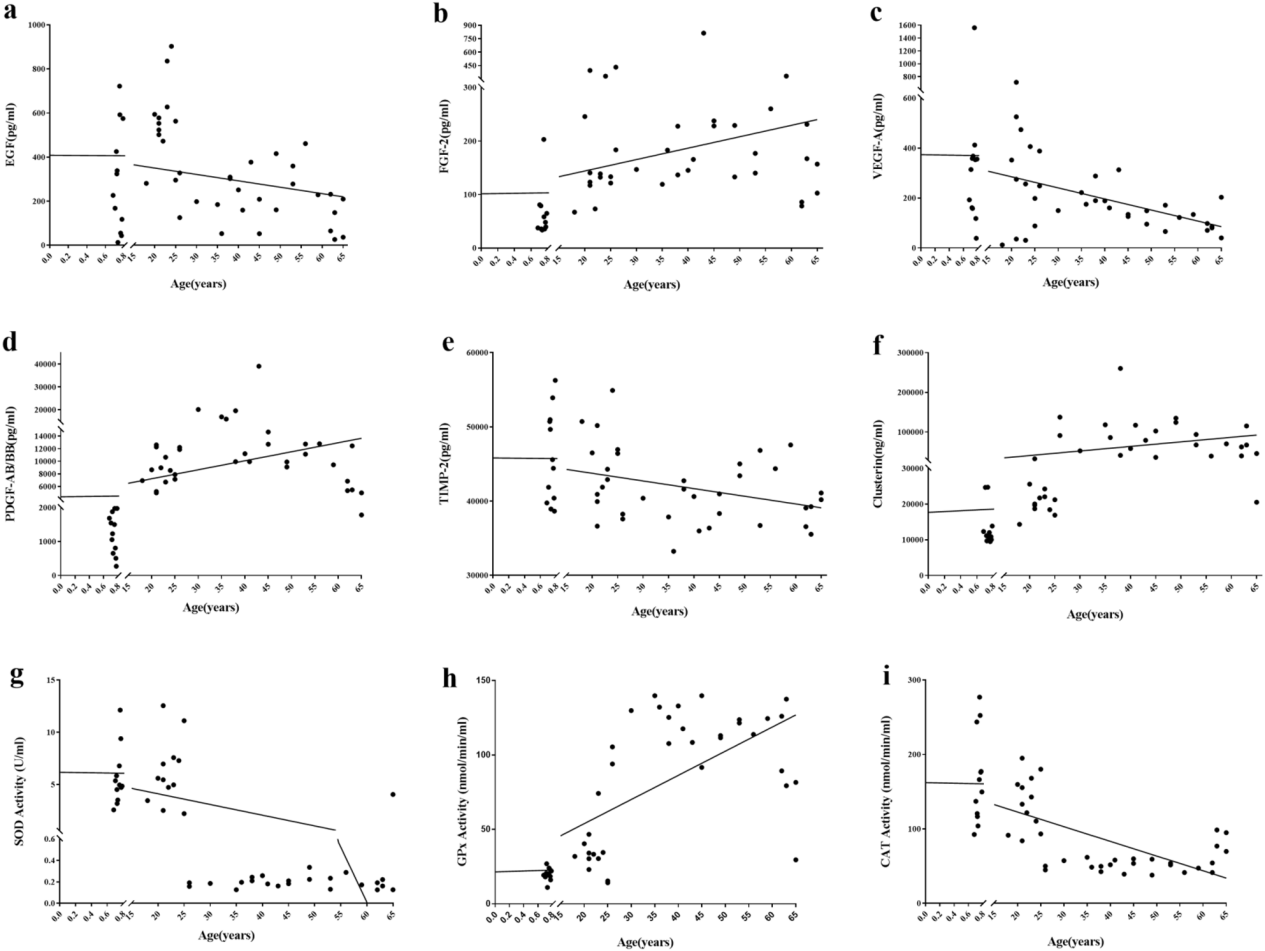
Scatter plots showing the correlation between the age of female participants who donated umbilical cord blood or peripheral blood and concentration of nine analytes in platelet-rich plasma (PRP) derived from those blood samples. (**a**) EGF: *r* =  − 0.285, *P* < 0.05; (**b**) FGF-2: *r* = 0.346, *P* < 0.05; (**c**) VEGF-A: *r* =  − 0.396, *P* < 0.01; (**d**) PDGF-AB/BB: *r* = 0.448, *P* < 0.01; (**e**) TIMP-2: *r* =  − 0.411, *P* < 0.01; (**f**) clusterin: *r* =  − 0.503, *P* < 0.01; (**g**) SOD:* r* =  − 0.634, *P* < 0.01; (**h**) GPx: *r* = 0.748, *P* < 0.01; (**i**) CAT: *r* =  − 0.695, *P* < 0.01.

As shown in Table 2 and Fig. 1, participant age showed a significant positive correlation with FGF-2, PDGF-AB/BB, and clusterin concentration and GPx activity (*P* = 0.016, 0.001, 0.000, and 0.000, respectively) and a significant negative correlation with EGF, VEGF-A, and TIMP-2 concentration and SOD and CAT activity (*P* = 0.049, 0.005, 0.004, 0.000, and 0.000, respectively).

**Table 2:** Pearson’s correlation coefficient analysis to determine the correlation between age of umbilical cord blood or peripheral blood donors and the concentration or activity of different bioactive components of platelet-rich plasma (PRP).

Variable	Group A (*n* = 12)	Group B (*n* = 12)	Group C (*n* = 12)	Group D (*n* = 12)	Pearson’s correlation with age	P
Age (years)	0.74 ± 0.03	22.00 ± 2.09	36.92 ± 6.65	58.25 ± 6.00	–	–
PLT (× 10^9^/L)	337.67 ± 60.780	307.5 ± 61.78	214.92 ± 32.38	261.83 ± 56.09	− 0.493	0.00**
WBC (× 10^9^/L)	6.48 ± 1.99	6.59 ± 1.62	6.82 ± 3.17	5.67 ± 1.59	− 0.100	0.51
IGF-1 (pg/mL)	19,268.75 ± 51,917.99	2241.83 ± 2517.11	324.26 ± 450.62	629.78 ± 1150.60	− 0.245	0.094
EGF (pg/mL)	300.11 ± 237.66	560.72 ± 180.80	212.62 ± 105.22	218.54 ± 142.56	− 0.285	0.049*
FGF-2 (pg/mL)	62.69 ± 47.37	168.42 ± 102.17	251.56 ± 195.07	174.53 ± 76.31	0.346	0.016*
VEGF-A (pg/mL)	366.07 ± 395.04	280.68 ± 222.42	215.41 ± 80.09	109.83 ± 47.49	− 0.396	0.005**
PDGF-AA (pg/mL)	5875.28 ± 3005.83	12,291.33 ± 3313.06	8727.17 ± 3596.95	6260.4167 ± 1499.04	− 0.053	0.722
PDGF-AB/BB (pg/mL)	1257.79 ± 595.33	8377.75 ± 2467.81	16,185.5 ± 8000.58	8494.67 ± 3588.78	0.448	0.001**
RANTES (pg/mL)	125,366.58 ± 85,548.04	184,753.75 ± 51,755.59	154,906.83 ± 49,305.29	130,228.1667 ± 72,719.14	− 0.069	0.642
TIMP-1 (pg/mL)	89,911.33 ± 32,069.20	139,087.08 ± 28,465.14	90,831.25 ± 14,181.49	109,439.92 ± 20,915.35	0.069	0.642
TIMP-2 (pg/mL)	45,927.17 ± 6195.48	45,186.08 ± 5154.36	38,664 ± 2718.60	41,302 ± 4097.44	− 0.411	0.004**
GDF11 (pg/mL)	128.47 ± 70.91	143.33 ± 101.94	202.47 ± 122.57	85.18 ± 38.45	− 0.106	0.472
Clusterin (pg/mL)	13.35 ± 5.44	21.29 ± 4.67	98.38 ± 60.24	73.39 ± 36.40	0.503	0.000**
SOD activity (U/mL)	5.65 ± 2.71	6.20 ± 3.16	0.19 ± 0.04	0.52 ± 1.11	− 0.634	0.000**
GPx activity (nmol/min/mL)	19.98 ± 4.07	34.0 ± 15.68	118.75 ± 17.02	104.32 ± 29.89	0.748	0.000**
CAT activity (nmol/min/mL)	167.71 ± 61.00	136.21 ± 36.74	51.38 ± 7.06	60.50 ± 20.39	− 0.695	0.000**

Values in this table were shown as mean ± standard deviation.

*PLT* platelets, *WBC* white blood cells.

*Represents *P* < 0.05.

**Represents *P* < 0.01.

**Figure 2:**
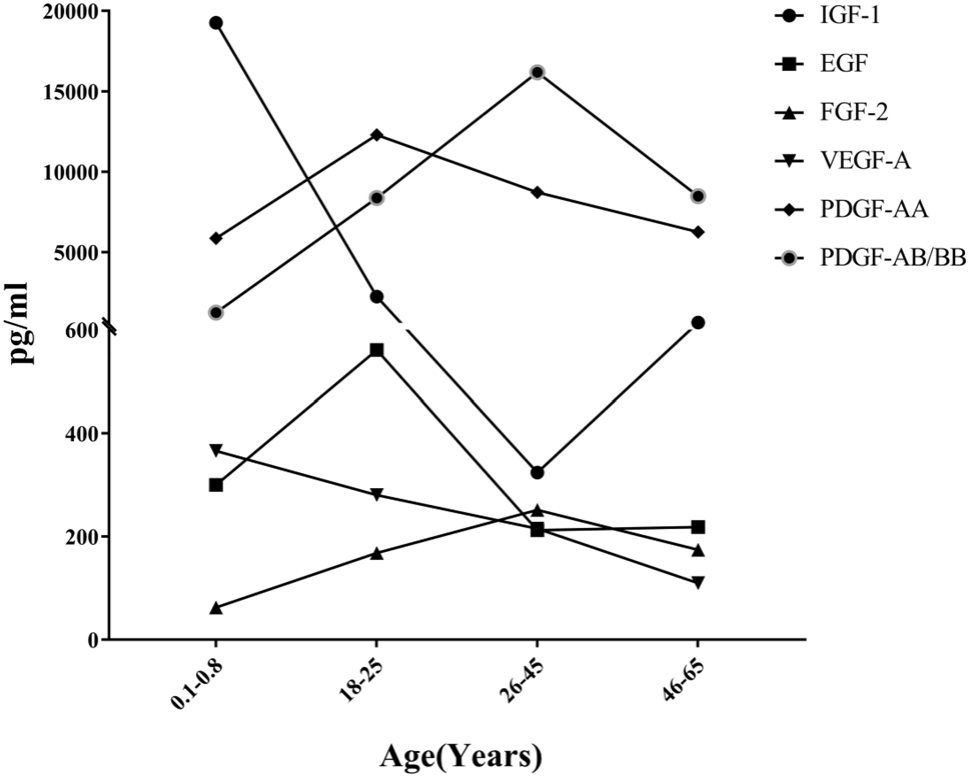
Relationship between the age of female participants who donated umbilical cord blood or peripheral blood and concentration of growth factors in platelet-rich plasma (PRP) derived from those blood samples. The concentrations in Group B and Group C are higher.

**Figure 3:**
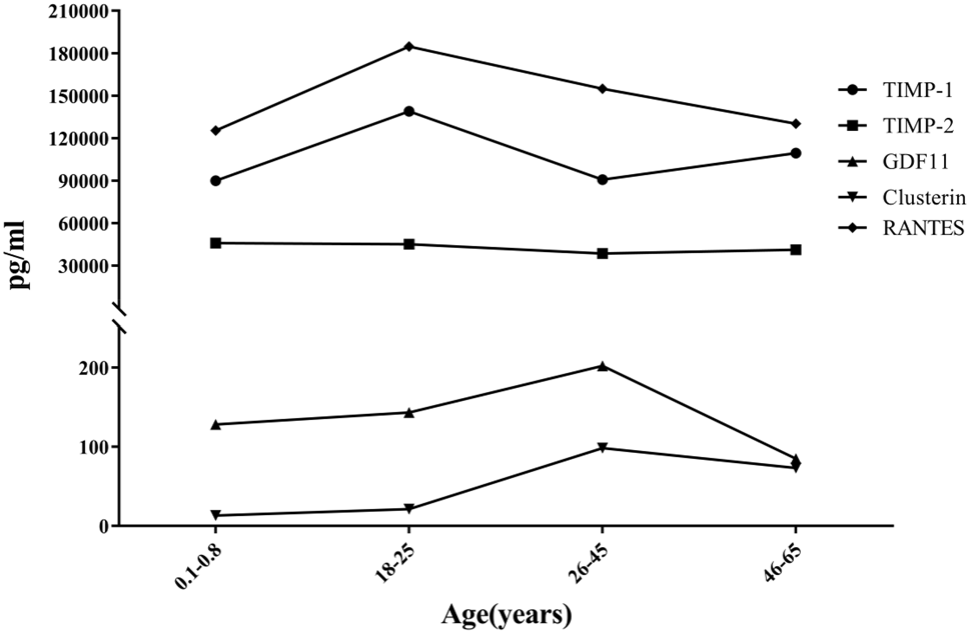
Relationship between the age of female participants who donated umbilical cord blood or peripheral blood and concentrations of anti-ageing-related proteins in platelet-rich plasma (PRP) derived from those blood samples. Anti-ageing proteins and endogenous antioxidant enzymes showed higher concentrations in Group A, Group B, and Group C.

**Figure 4:**
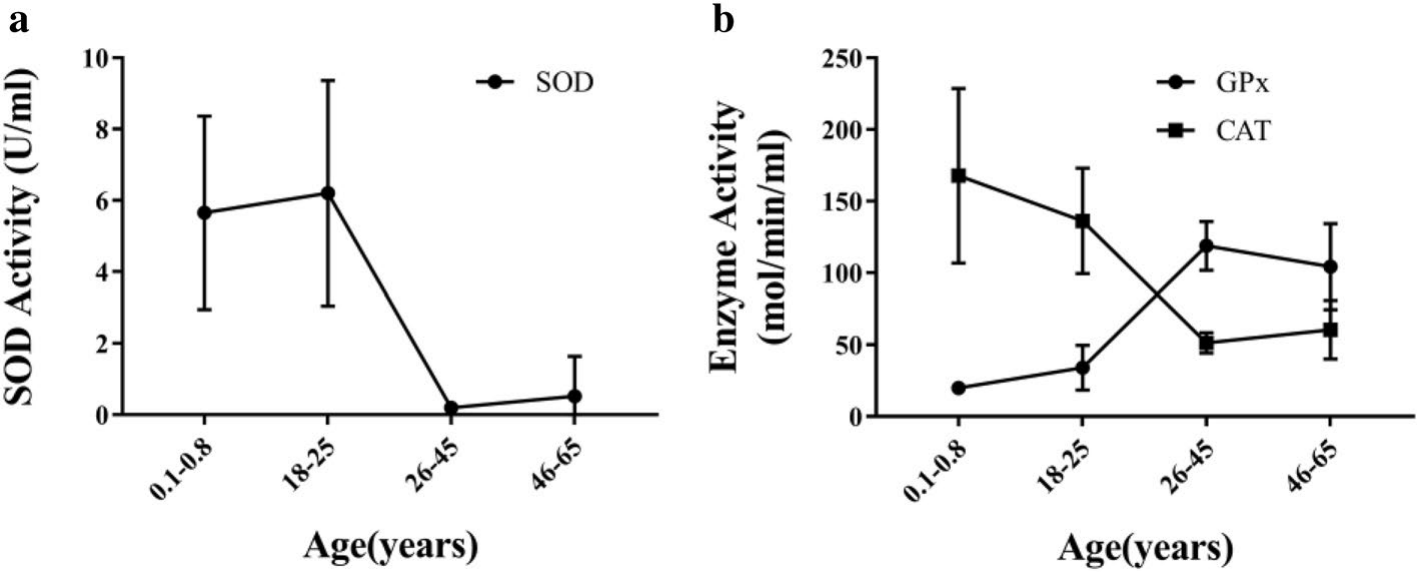
Relationship between the age of female participants who donated umbilical cord blood or peripheral blood and concentrations of endogenous antioxidant enzymes in platelet-rich plasma (PRP) derived from those blood samples. The levels of CAT, SOD, and GPx were the highest separately in Group A, Group B, and Group C, respectively.

**Figure 5:**
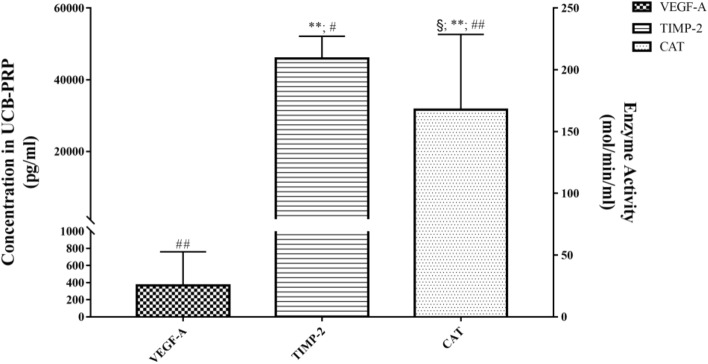
Analytes with a significantly higher concentration or activity in platelet-rich plasma derived from umbilical cord blood (UCB-PRP) than PRP derived from peripheral blood from adult females. ^§^Represents *P* < 0.05 vs. Group B, **represents *P* < 0.01 vs. Group C; ^#^represents *P* < 0.05 vs. Group D, ^##^represents *P* < 0.01 vs. Group D. The bioactive components with a significantly higher concentration/activity in UCB-PRP were VEGF-A, TIMP-2, and CAT.

We analysed the differences in the concentrations of various bioactive components (Fig. 2), anti-ageing proteins (Fig. 3), and endogenous antioxidant enzymes (Fig. 4) in PRP derived from females belonging to different age groups. We observed that each group, except Group D, showed significantly higher concentrations of certain components than the other groups. The concentrations of GFs were higher in Group B and Group C. The concentrations of anti-ageing proteins and endogenous antioxidant enzymes were higher in Group A, Group B, and Group C. The concentration of most active ingredients in Group D was high but not significantly different from that in the other groups. VEGF-A and TIMP-2 levels and CAT activity in Group A were observed to be significantly higher in some pairwise comparisons (Fig. 5).

## Discussion

Since the 1970s, PRP has been widely used in clinical settings because it secretes GFs and proteins that promote tissue repair and regeneration^23^. Additionally, PRP has been reported to enhance the recruitment, proliferation and differentiation of cells involved in tissue regeneration^24^. Platelet-derived GFs, the earliest known components of PRP, play an important role in cell proliferation, chemotaxis, cell differentiation, regeneration, and angiogenesis^25^. It is generally believed that active substances such as GFs are negatively affected by ageing. Previous studies that investigated the relationship between age, gender, and GFs in PRP reported that only a few GFs were significantly correlated with age and gender. Some studies reported a weak negative correlation between age and levels of PDGF-AB/BB and IGF-1, but these studies did not provide more details about purified PRP purification nor did they perform a gender analysis^15–17,26,27^.

PDGF is an effective mitogen and chemokine produced by mesenchymal-derived cells. High-affinity cell-surface receptors specific for PDGF have been reported only on connective tissue cells. PDGF has been demonstrated to stimulate the proliferation of connective tissue cell types, such as fibroblasts, in vitro and regulate the production of pro-inflammatory and anti-inflammatory mediators and tissue permeability and hemodynamics^27^. In muscle precursor cells, PDGF-AB/BB markedly promoted proliferation and inhibited differentiation in vitro, contributing to skin regeneration^28^. In the present study, we found a positive correlation between donor age and FGF-2 and PDGF-AB/BB levels, and the highest concentrations of both were consistently found in group C (females aged 26–45 years). This finding indicates that PRP derived from females in this age group may be more effective in clinical treatment of muscle and tendon diseases.

Clusterin, a multifunctional secreted glycoprotein, protects retinal vascular endothelial cells and retinal pigment epithelial cells, reduces cell apoptosis, promotes corneal epithelial cell proliferation, and induces astrocyte and neuron differentiation^29^. Previous studies have reported that autologous PRP can be used for the treatment of diabetic xerophthalmia and relieves the symptoms of diabetic xerophthalmia, such as dryness, itching, redness, and burning^30^. In the present study, clusterin levels was found to be positively correlated with age (*P* = 0.0003). This finding suggests that clusterin, as an important bioactive component of PRP used as eye drops, may promote corneal regeneration and that autologous PRP is a better treatment choice to relieve eye dryness and discomfort in women aged 26–45 years.

Contrary to expectation, UCB-derived PRP did not exhibit a noteworthy bioactive component profile: the levels of bioactive components released from UCB-derived PRP were not significantly higher than those in other groups, except for VEGF-A and TIMP-2. This finding may be attributed to the low reactivity of neonatal platelets, which exhibit diminished responses to platelet agonists than adult platelets, resulting in lower granule secretion, fibrinogen binding, and platelet aggregation; this hyporesponsive phenotype persists for several weeks after birth^6,27,28^. Platelet activation requires agonists to stimulate multiple G-protein-coupled receptors, leading to rapid mobilization of calcium, α- and dense-granule release, cytoskeletal recombination, thrombin A2 release, and conformational changes in the glycoprotein IIb/IIIa complex to promote platelet aggregation^1^. Previous studies have demonstrated that neonatal and UCB-derived platelets exhibit reduced PAR1-mediated particle secretion and integrin activation than adult platelets and are associated with lower PAR1 expression of neonatal thrombocytopenia^31^. Previous studies have reported that the levels of PDGF-AB and transforming growth factor-β1 in UCB-derived PRP were equivalent to or slightly higher than those in PRP derived from peripheral blood^8^.

However, in our study, we also observed that the bioactive components with the highest level in UCB-derived PRP: VEGF-A, CAT activity and TIMP-2 (the difference was statistically significant), among which the anti-ageing protein TIMP-2 was worthy of attention. Studies have shown that plasma derived from human UCB or blood from young donors is a rich source of TIMP-2 and GDF11 and can be used to reverse the effects of certain age-related diseases^6,7,31,32^. In the present study, a high concentration of TIMP-2 was found in UCB-derived PRP, which was significantly different from that in two other groups (Group A vs. Group C, *P* = 0.00; Group A vs. Group D, *P* = 0.02), and TIMP-2 concentration was negatively correlated with donor age. This finding demonstrates the potential significance of further exploring the anti-ageing effects of UCB-derived PRP. Nevertheless, GDF11, previously reported to be an anti-ageing protein, did not show a significantly higher concentration in Group A or a significant correlation with donor age.

Oxidative stress plays an important role in the development of vascular diseases, which affect vital organs, particularly the brain and heart. ROS have significant toxic and harmful effects owing to their high reactivity. In general, ROS production increases with ageing^33^, and high ROS levels with the subsequent oxidative stress are associated with pathological conditions^34^. The presence of antioxidant enzymes such as SOD, CAT, and GPx maintains normal levels of ROS homeostasis and minimizes the level of cellular stress. Notably, in the present study, we found that the levels of CAT, SOD and GPx were the highest in Group A, Group B, and Group C, respectively, and GPx activity was positively correlated with age. However, the levels of antioxidant enzymes in PRP are considerably lower than those in whole blood, and the activity of antioxidant enzymes in PRP needs to be further studied^35,36^.

This study intended to exclude the effects of differences in oestrogen levels between females and males. Research has shown that the higher levels of oestrogens in females upregulate the expression of antioxidant and longevity-related genes, such as selenium-dependent GPx and Mn-SOD, which play a role in anti-ageing^37^. Gender differences have also been linked to variability in wound repair: androgens are believed to hamper healing, whereas oestrogen has a beneficial effect on healing^38,39^. Oestrogen is associated with an increase in TGFb-1 and regulates VEGF and IGF-1 gene expression^40–42^. Consistent with these findings, research has also demonstrated a diminished healing response in post-menopausal women^40^. In the present study, PRP derived from blood obtained from women older than 45 years (group D) did not contain significantly higher concentrations of any of the tested components than PRP in other groups, further suggesting that age and ageing significantly affect the active components of PRP. In the future, the selection of clinical treatment with autologous PRP in elderly female patients needs more careful consideration.

Nevertheless, the present study also has certain limitations. We investigated the levels of active substances secreted by platelets in PRP obtained from females, but this study did not include the relevant data on oestrogen levels, and our data do not provide information for the male population. In addition, the biological characteristics of UCB are affected by multiple obstetric factors such as the size of the baby, placental weight, gestational age, and delivery mode^43^. Thus, this study only preliminarily demonstrates the active components secreted by platelets from umbilical cord blood, and the specific role of UCB-PRP needs to be further explored. Moreover, the participant age range was limited; the present findings cannot be extended to individuals over 65 years old. Although we showed that the variance in the levels of certain anti-ageing components of PRP were significantly correlated with age, further studies are necessary to determine the clinical significance of these findings. The results of this study are mostly exploratory in nature; therefore, the findings should be interpreted with caution.

## Conclusion

The present findings suggest that the concentration of bioactive components of PRP derived from human UCB from female neonates and peripheral blood from adult females was not significantly different. With increasing age, the concentration of some biologically active substances in PRP decreases. PRP derived from UCB contains various active ingredients including anti-ageing proteins. Owing to its rich source of bioactive components and low immunogenicity, the use of UCB to prepare PRP is an important research direction for future studies.

## Data Availability

The datasets used and/or analysed during the current study available from the corresponding author on reasonable request.
